# Transient biological response of human mesenchymal stem cells to a single wideband high-power electromagnetic pulse exposure: a preliminary study

**DOI:** 10.3389/fcell.2026.1873951

**Published:** 2026-07-02

**Authors:** Alicja Trębińska-Stryjewska, Monika Dobrzyńska, Rafał Bogdan Lewandowski, Wiktoria Kasprzycka, Małgorzata Stępińska, Paulina Natalia Osuchowska, Łukasz Paweł Osuchowski, Jacek Starzyński, Zygmunt Mierczyk, Elżbieta Anna Trafny

**Affiliations:** 1 Biomedical Engineering Centre, Institute of Optoelectronics, Military University of Technology, Warsaw, Poland; 2 Institute of Electronic Systems, Faculty of Electronics, Military University of Technology, Warsaw, Poland

**Keywords:** cell cycle, gene set enrichment analysis, high-power electromagnetic pulse, mesenchymal stem cells, mitochondrial superoxide, oxidative phosphorylation

## Abstract

This preliminary study examines the effects of a single wideband high-power electromagnetic (HPEM) pulse on human bone marrow-derived mesenchymal stem cells (hBM-MSCs). The exposure was designed to emulate a realistic electromagnetic exposure scenario involving a magnetocumulative generator, a non-repetitive but extremely high-energy source. The pulse, characterized by a peak field of 1 MV/m, 120 ns pulse width, and 50 MHz center frequency, was applied to hBM-MSCs primary cell lines. We assessed cellular morphology, viability, proliferation, mitochondrial function, oxidative stress, DNA damage, and gene expression at various time points post-exposure. Our findings indicate no observable changes in cell morphology, viability, or proliferation compared to control and sham-treated cells. Mitochondrial membrane potential was unaffected by exposure; however, a consistent increase in mitochondrial superoxide production was observed, with a main effect of treatment across both cell lines. DNA damage assessments revealed no detectable induction of single- or double-strand breaks. Gene set enrichment analysis identified transient upregulation in gene sets related to cell cycle progression and oxidative phosphorylation immediately post-exposure, though these effects were not sustained. Overall, a single HPEM pulse did not cause lasting detrimental effects on hBM-MSCs. However, subtle and transient biological responses were observed, highlighting the need for further investigation into the effects of electromagnetic pulses on human cells.

## 1 Introduction

Due to their ability to interfere with or even damage electronic equipment, high-power electromagnetic (HPEM) pulses are of increasing interest to military and law enforcement agencies. One of the most promising applications of this technology would be to neutralize unmanned aerial vehicles (drones) (Razooqi and Ali, 2022). On the ground, HPEM pulse generators could be used to control speeding vehicles, support checkpoint operations, protect convoys and defend infrastructure. They could also be useful in disabling electronic triggers of improvised explosive devices (Hong and Braidwood, 2010; Urbancokova et al., 2015).

For HPEM pulse-generating devices, the level of exposure in the far field (far from the antenna) is well defined, whereas in the near field (Fresnel zone) it is more difficult to describe accurately due to the presence of reflections. As a result, occupational exposure of the personnel operating such equipment may be underestimated (Miclaus et al., 2016). The thermal effects of electromagnetic radiation on living organisms have been extensively studied, and standards have been established based on these findings. In contrast, the non-thermal effects of HPEM pulses remain relatively poorly understood (Mukhopadhyay and Sanyal, 1997; Schunck et al., 2014).

There are relatively few studies investigating the biological effects of nanosecond wideband and ultra-wideband HPEM (WB/UWB HPEM) pulses without accompanying temperature rise in mammals and mammalian cells. They also provide some contradictory findings. Most of the animal studies showed no effects on animal behavior, development, blood chemistry, cardiovascular and nervous systems, vascular permeability or the tumor growth rate in rats and mice (Schunck et al., 2014; Kolosnjaj-Tabi et al., 2021). However, there are also reports on temporary liver and renal disfunction in mice accompanied with increased levels of oxidative stress (Guo et al., 2020), transient increase in blood-brain barrier permeability (Ding et al., 2010; Gao et al., 2020) or neuronal abnormalities associated with elevated levels of oxidative stress and apoptosis in neurons (Deng et al., 2014). At the cellular level, WB/UWB-HPEM pulses either had no effect or increased the proliferation rate of pre-neoplastic CL-S1 mammary epithelial cells, when the cells were exposed to a higher number of pulses and in the presence of a mitogen (Sylvester et al., 2005). Similarly, pulsed WB/UWB-HPEM had a mitogenic effect on mouse AML-12 hepatocytes, especially in the presence of growth factors (Dorsey et al., 2005). In contrast, Gibot and colleagues (Gibot et al., 2019) found that there were no differences in the growth of spheroids, composed of either normal human fibroblasts or HCT-116 colon carcinoma cells when exposed to a different number of WB/UWB-HPEM pulses. Exposure did not result in the permeabilization of these cells, in contrast to the pulsed electric field (Pillet et al., 2018). Other effects included the formation of heterochromatin granules, which are indicative of cellular stress, in human buccal epithelial cells after exposure (Shckorbatov et al., 2009), and increased DNA binding of NFKB transcription factor in human monocytes without resulting changes in gene expression (Natarajan et al., 2006).

So far, only one preliminary study on mesenchymal stem cells exposed to WB-HPEM pulses has been published by Czwartos and colleagues (Czwartos et al., 2023). No effects of the exposure were reported. Adult stem cells are a very interesting model to study due to their ability to self-renew and differentiate (Ullah et al., 2015), as well as to modulate immune response, angiogenesis, cell death and tissue regeneration through their secretome, consisting of both soluble molecules (e.g., cytokines, growth factors) and factors released in extracellular vehicles (Trigo et al., 2025). Extremely low frequency pulsed electromagnetic fields (frequencies of 5–75 Hz, magnetic field intensities of 0.001–7 mT) have been shown to enhance MSCs proliferation and osteogenic differentiation (Sun et al., 2009; Ongaro et al., 2014; Kim et al., 2015; Ross et al., 2015) as well as their chondrogenic potential (Varani et al., 2021), with an important role of MSCs’ secretome in stimulating chondrogenesis, enhancing migration, and decreasing expression of inflammatory markers (Parate et al., 2020). Therefore, we asked whether high-power electromagnetic pulses with a center frequency in a range of MHz could influence the biological processes in hBM-MSCs. The answer to this question may also be relevant for occupational safety, public exposure assessment, and health risk evaluation related to high-intensity electromagnetic environments. This study presents preliminary results that will aid in the design of future confirmatory investigations.

## 2 Materials and methods

### 2.1 Cell culture

Two cell lines of PoieticsTM Normal Human Bone Marrow Derived Mesenchymal Stem Cells (hBM-MSC) (PT 2501, Lonza, Basel, Switzerland) from different donors were propagated in MSCGM Mesenchymal Stem Cell Growth Medium BulletKit (Lonza), and antibiotic/antimycotic in 5% carbon dioxide at 37 °C and 90% humidity until semi-confluent. Per manufacturer’s information, cell line no. 1 (hBM-MSC1, lot number 0000588695) was obtained from a 22-year-old male of Hispanic/Latino origin, and cell line no. 2 (hBM-MSC2, lot number 18TL169252) was isolated from a 24-year-old Black female. The purchased cells were attested to differentiate down the adipogenic, chondrogenic, and osteogenic lineages when cultured in the recommended differentiation medium, to express CD29, CD44, CD73, CD90, CD105, and CD166 (≥90% positive) and to not express CD14, CD19, CD34, CD45, and HLA-DR (≤10% positive).

### 2.2 HPEM exposure

The overall experimental design included two distinctive hBM-MSC lines, with two independent experiments performed on separate days for each cell line, and three technical replicates for each condition (exposed, sham-exposed and control) in every experiment. Human BM-MSCs were used for experiments at the fourth or fifth passage. They were cultured for 48–72 h in separate 175 cm^2^ cell culture flasks (unless otherwise stated) before reaching 80% confluence. Cell culture flasks were then randomly assigned to the exposed, sham-exposed or control group using a simple randomization method. Prior to the exposure, the standard hBM-MSC medium was replaced with 10 mL of fresh, warm medium.

Cell culture flasks representing technical replicates were simultaneously exposed to a single unipolar wideband HPEM pulse (peak field: 1 MV/m, rise time: 3 ns, pulse width: 120 ns, center frequency: 50 MHz) inside a UCC-L Frankonia anechoic chamber. A detailed technical description of the system used to generate HPEM pulses has been published previously (Czwartos et al., 2023). That publication provides a comprehensive account of the pulse generation setup and the monitoring of its parameters, including a time-domain profile of the measured electric field and an analysis of the specific absorption rate (SAR) delivered to the probe. In brief, a 600 kV Marx generator discharged into a stripline, producing the electric field, which was measured using a D-dot probe. The SAR associated with a single pulse was negligible; therefore, the primary factor influencing the cells was the intense, rapidly rising electromagnetic field. The variability of the exposure signal across experimental sequences was low: the rise time ranged from 3.0 to 4.5 ns, the pulse width remained stable within 9% at half-maximum, and the peak field amplitude varied between 944 and 1018 kV/m.

Sham-treated cells were prepared as above, except that they were placed in a Faraday cage (80 dB attenuation for frequencies ranging from 100 kHz to 1 GHz) during exposure. Control cells were maintained in standard hBM-MSC culture conditions prior to harvesting.

After the treatment, cells were harvested immediately or cultured for 4 h or 24 h following the addition of 40 mL of standard hBM-MSC medium. Cell detachment was performed enzymatically using the Lonza ReagentPack Subculture Reagents (CC-5034), recommended for primary cell subculturing including human BM-MSCs. Briefly, cells were washed with HEPES Buffered Saline and incubated with Trypsin/EDTA until they detached from the surface (approximately 5 min). Trypsin/EDTA was then inhibited by Trypsin Neutralizing Solution. The subsequent processing of the samples is described for each method.

### 2.3 Optical microscopy

Cells collected immediately, 4 h or 24 h after treatment were seeded into 24-well plates (4-8×10^5^ cell/mL, 1 mL) and allowed to adhere for 1 h. The morphology of the control, sham, and exposed cells was observed using an inverted light optical microscope (Primo Vert, Carl Zeiss, Oberkochen, Germany) equipped with ZEISS ZEN software (Carl Zeiss).

### 2.4 Scanning electron microscopy (SEM)

Cells were prepared similarly to light microscopy, except they were seeded onto glass slides in 24-well plates and allowed to adhere for 2 h before fixation. The cells were fixed with a solution of 4% formaldehyde and 0.4% glutaraldehyde, followed by an overnight incubation in 1% osmium tetroxide. The cells were then dehydrated through a series of ethanol solutions with increasing concentrations (30%, 50%, 70%, 80%, 90%, 96%, and 99.9%) and acetone (30%, 50%, and 100%). The samples were subsequently dried using a critical point dryer (EM CPD300, Leica, Wetzlar, Germany) and coated with a sputter coater (EM ACE200, Leica). Finally, imaging was performed using a scanning electron microscope (STEM, Quanta FEG250, FEI, Hillsboro, OR, United States of America) in high vacuum mode with an Everhart-Thornley detector (ETD).

### 2.5 LIVE/DEAD cell viability assay

The LIVE/DEAD™ Viability/Cytotoxicity Kit for mammalian cells (Invitrogen, Waltham, MA, United States of America) was employed to evaluate cell viability. Cells were cultured in 24-well plates at a density of 1 × 10^5^ cells per well, with separate plates prepared for the exposed, sham-treated, and control groups. After a 24-h incubation period, the medium was replaced with 223 µL of fresh medium. Following the exposure, all plates underwent the same treatment. For each well, 1 mL of a solution containing 4 µM ethidium homodimer-1 (EthD-1) and 0.5 µM calcein-AM in pre-warmed PBS was added. The cells were incubated for 15 min at 37 °C in the dark and then imaged using a confocal fluorescence microscope (LSM 700 Axio Observer. Z1, Carl Zeiss).

### 2.6 Presto blue cell metabolic activity assay

Metabolic activity was assessed using the PrestoBlue Cell Viability Reagent (Molecular Probes, Invitrogen). Following exposure, the cells were seeded into 96-well flat-bottom transparent culture plates (Greiner, Kremsmünster, Austria). The PrestoBlue assay was performed 24 h after exposure according to the manufacturer’s instructions. Samples were incubated with the PrestoBlue reagent for 2 h in the dark at 37 °C in a humidified atmosphere with 5% CO_2_. Standard hBM-MSC medium with PrestoBlue was used as a blank control. Fluorescence intensity was measured using a CLARIOstar microplate reader (BMG LABTECH, Ortenberg, Germany). End-point fluorescence readings were taken at 593 ± 10 nm following excitation at 557 ± 10 nm, using a dichroic mirror at 498.8 nm, with top optic reading and gain set to 1290.

### 2.7 CyQuant cell proliferation assay

Cell proliferation was evaluated using the CyQuant NF Cell Proliferation Assay (Invitrogen), which employs a fluorescent DNA-binding dye. Exposed, sham-exposed, and control cells were plated in 96-well plates (Greiner). The CyQuant assay was conducted 24 h after exposure, following the manufacturer’s protocol. Samples were incubated with CyQuant GR dye for 1 h in the dark at 37 °C within a 5% CO_2_ environment. A blank control containing medium and CyQuant GR dye, but no hBM-MSC cells, was also included. Fluorescence intensity was assessed using a CLARIOstar microplate reader (BMG LABTECH). End-point fluorescence measurements were recorded at 530 ± 20 nm after excitation at 485 ± 15 nm, using a dichroic mirror set at 506.2 nm, with a top optic reading and a gain adjusted to 926.

### 2.8 Mitochondrial membrane potential ΔΨm detection

The Mitochondria Staining Kit for Detecting Changes in Mitochondrial Potential (Sigma-Aldrich, Burlington, MA, United States of America) was utilized according to the manufacturer’s protocol. Cells were harvested either immediately or 4 h post-treatment, and the cell pellets were resuspended in 1 mL of culture medium. A 1:1 mixture of the cell suspension and a buffer (1× staining buffer) containing JC-1 dye at a concentration of 5 μg/mL was prepared. The samples were stained for 20 min at 37 °C in an incubator with 5% CO_2_ and protected from light. For positive control, 1 µL of a 1 mg/mL valinomycin solution was added to the cell suspension. After staining, the samples were centrifuged at 600×g at 3 °C and washed with cold 1× staining buffer. The cell pellets were then resuspended in 0.5–1 mL of cold 1× staining buffer. Samples were kept on ice during cytometric analysis. Following excitation at 488 nm, fluorescence emission intensities were recorded in the green (530/30 nm) and yellow (585/15 nm) channels using a BD FACSAria III (Beckton, Dickinson and Company, NJ, United States of America) with BD FACSDiva software 10.0 (Beckton, Dickinson and Company). The ratio of yellow to green fluorescence was subsequently calculated.

### 2.9 Measurement of oxidative stress in living cells

Oxidative stress in living cells was evaluated using two fluorescent probes: the cytoplasmic reactive oxygen species indicator CellROX Orange Reagent (Invitrogen) and the MitoSOX Red Mitochondrial Superoxide Indicator (Invitrogen). The assays were conducted according to the manufacturer’s instructions. After treatment, the cells were transferred to 96-well plates (Greiner), gently centrifuged at 70×g for 3 min, and incubated at 37 °C in a 5% CO_2_ environment to allow attachment. Following a 30-min incubation, the medium was replaced with a warm PBS (Phosphate-Buffered Saline) solution containing 5 µM of either CellROX Orange or MitoSOX Red. The cells were then incubated in the dark at 37 °C for 30 min (for CellROX Orange) or 10 min (for MitoSOX Red). Fluorescence intensity was measured using a CLARIOstar microplate reader (BMG LABTECH) with the following settings: for CellROX Orange, excitation at 550 ± 25 nm, emission at 605 ± 40 nm, dichroic mirror at 572.5 nm, top optic reading, and gain set to 2951; for MitoSOX Red, excitation at 525 ± 15 nm, emission at 615 ± 20 nm, dichroic mirror at 568.8 nm, top optic reading, and gain set to 3000.

### 2.10 Apoptosis detection

Apoptosis was evaluated using two fluorescent dyes: annexin V-Alexa Fluor 488 (Annexin V) and propidium iodide (PI), immediately following exposure of the cells to HPEM. Control and sham-exposed cells were used as negative controls. The analysis was conducted on a BD FACSAria III flow cytometer (Beckton, Dickinson and Company) equipped with a 488 nm argon laser, and data was processed using BD FACSDiva software version 10.0 (Beckton, Dickinson and Company). For each sample, a minimum of 10^4^ cells were recorded, and all samples were run in triplicate. Gating was defined in the green 530/30 channel for Alexa Fluor 488 and the yellow 585/45 channel for PI, with cells categorized as follows: live cells (Annexin V negative, PI negative), apoptotic cells (Annexin V positive, PI negative), late apoptotic/necrotic cells (Annexin V positive, PI positive), and dead cells (Annexin V negative, PI positive).

### 2.11 Comet assay

The OxiSelect Comet Assay Kit (Cell Biolabs, San Diego, CA, United States of America) was employed according to the provided instructions. After treatment, hBM-MSCs at a concentration of 1 × 10^5^ cells/mL were washed and mixed with 10% low melting point OxiSelect Comet Agarose. Then, 75 μL of this cell/agarose suspension was pipetted into each well of the OxiSelect 3-Well Comet Slides. These slides were kept in the dark at 4 °C for 15 min to allow the agarose to solidify. Following this step, the slides were submerged in cold lysis buffer for 60 min at 4 °C, also in the dark. After lysis, the buffer was replaced with pre-chilled alkaline buffer, and the slides were incubated at 4 °C for another 30 min. The slides were then rinsed twice with cold TBE (Tris-borate-EDTA) buffer for 5 min each, after which they underwent electrophoresis at 1.3 V/cm for 20 min. Comets stained with ethidium bromide (20 μg/mL) were viewed using a Nikon Eclipse Ci-L epifluorescence microscope (Tokyo, Japan), and images of at least 70 cells per slide were captured for each experimental group. As a positive control to assess DNA damage, hBM-MSC cells were treated with 50 µM etoposide (Sigma-Aldrich) for 24 h. DNA damage levels were analyzed using CaspLab software (http://casplab.com, 2020). To measure the extent of DNA damage, the percentage of DNA in the comet tail and the tail moment (defined as the percentage of DNA in the tail multiplied by tail length) were calculated. Additionally, the nuclei were categorized into five groups based on the DNA content in the comet tail, following the classification method described by Focke et al. (Focke et al., 2010).

### 2.12 RNA isolation

After collection, the cells were rinsed twice with ice-cold PBS and centrifuged at 4 °C for 5 min at either 150×g (hBM-MSC1) or 300×g (hBM-MSC2). The cell pellets were then stored at −80 °C until RNA extraction. Total RNA was extracted using the RNeasy Mini Kit (QIAGEN, Hilden, Germany), following the manufacturer’s instructions, which included DNA digestion on the column with DNase I. RNA concentration was measured using the QuantiFluor RNA System (Promega, Madison, WI, United States of America) and the Quantus Fluorometer (Promega). RNA quality was evaluated with the 2100 Agilent Bioanalyzer and Agilent RNA 6000 Nano Reagents (Agilent, Santa Clara, CA, United States of America), resulting in RNA Integrity Numbers (RIN) between 9.2 and 10.0.

### 2.13 Microarrays

Cyanine-3 (Cy3)-labeled cRNA was prepared from 100 ng total RNA as previously described (Kasprzycka et al., 2021; Kasprzycka et al., 2021) with the addition of RNA Spike-In, One Color (Agilent) as an internal control. Low Input Quick Amp Labeling Kit, One Color (Agilent) and RNeasy Mini Kit (QIAGEN) were used for this procedure according to the manufacturer’s instructions. The quality and quantity of cRNA were evaluated using an Agilent 2100 Bioanalyzer and Agilent RNA 6000 Nano Reagent. Cy3-labeled cRNA (600 ng) was fragmented and hybridized to SurePrint G3 Human Gene Expression v3 8 × 60 K (G4851C) microarrays at 65 °C for 17 h. Prior to scanning, microarrays were washed with GE Wash Buffer 1 and 2 (Agilent) according to the manufacturer’s instructions. Microarrays were scanned using the SureScan Microarray Scanner (Agilent), and data were extracted from the scanned images using Feature Extraction Software 12.0 (default parameters; protocol: GE1_1200_Jun14, grid: 072363_D_F_20190214).

### 2.14 Microarrays data analysis

GeneSpring 14.9 was used to process microarray data; processing included *log*_*2*_ transformation, normalization by percentile shift (75^th^ percentile), and baseline transformation (median of all samples). Processed and raw data are available in the NCBI’s Gene Expression Omnibus (Edgar et al., 2002; Edgar et al., 2002) database under GEO Series accession number GSE278937 https://www.ncbi.nlm.nih.gov/geo/query/acc.cgi?acc=GSE278937). Genes were filtered according to probe flag values and only probes detected in 100% of samples from a given condition were included. The following analyses were performed using GeneSpring 14.9: Principal Component Analysis (PCA) with four principal components, and differential gene expression analysis using a moderated t-test (p < 0.05) with Benjamini-Hochberg FDR multiple testing correction.

In addition, gene set enrichment analysis was done for HPEM-exposed and sham-exposed pairs using GSEA software (version 4.2.3) (Mootha et al., 2003; Subramanian et al., 2005) with hallmark gene sets (version 7.5.1) from the Molecular Signatures Database 2022, (https://www.gsea-msigdb.org/gsea/msigdb/index.jsp) (Liberzon et al., 2015). Only probes detected in 100% of samples from at least one condition were analyzed to exclude genes with ambiguous or no expression in hBM-MSCs. The main GSEA settings were as follows: probes for the same gene were collapsed (collapsing mode: median of probes), statistical significance was calculated with gene set permutation (no. of permutations: 1000), gene set size was between 15 and 500 genes, the false discovery rate (FDR) cut-off was set to 0.25. For gene sets with FDR<0.25, Normalized Enrichment Scores (NES) from GSEA were visualized on a heatmap generated with GraphPad Prism v10 (GraphPad Software LLC, La Jolla, CA, United States of America). Hallmark gene sets were grouped by a process category (Liberzon et al., 2015).

Leading edge analysis in GSEA was used to extract leading edge gene subsets from the enriched hallmark gene sets with FDR<0.25. Signal2Noise values, expressed as the difference of means scaled by the pooled standard deviation *(µE-µS)/(σE+σS)*, were extracted for each identified gene. Leading edge genes were compared between both cell lines at each time point, separately for positively enriched and negatively enriched gene sets. Identified common genes were subjected to protein-protein interaction network functional enrichment analysis using STRING version 11.5 (https://string-db.org/, 2024) (Jensen et al., 2009) with the following settings: type of network–full STRING network, nodes–genes, edges - confidence of interaction (high confidence: 0.7). Functional enrichment network analysis was also performed with STRING.

### 2.15 Statistical analysis

Identification and rejection of outliers in fluorescence intensity measurements (Presto Blue, CyQuant, CellROX Orange, and MitoSOX Red assays) was performed using Dixon’s Q test. Quantitative data from CyQuant, Presto Blue, CellROX and MitoSOX assays were analyzed using a Two-way Repeated Measures ANOVA to evaluate the impact of treatment groups (Control, Sham-control, and Exposed) across different cell lines. To account for the inherent biological variability between primary cell lines and technical variation between independent experiments, a matching design was employed where each experiment within a cell line was treated as a matched unit (block). Technical replicates were averaged within each independent experiment prior to analysis. Sphericity was not assumed; therefore, the Geisser-Greenhouse correction was applied to adjust the degrees of freedom. Following a significant main effect of treatment, post-hoc comparisons were done using Tukey’s multiple comparisons test to determine differences between specific groups. Statistical analysis was performed in GraphPad Prism v10.0.2 (GraphPad Software LLC). For the comet assay, Student’s t-test and non-parametric Mann-Whitney U test were used to compare two groups; these data were analyzed using Statistica version 13 (TIBCO Software Inc., Palo Alto, CA, United States of America). Statistical significance was set at (α < 0.05).

## 3 Results

### 3.1 Exposure conditions and hBM-MSCs morphology

**FIGURE 1:**
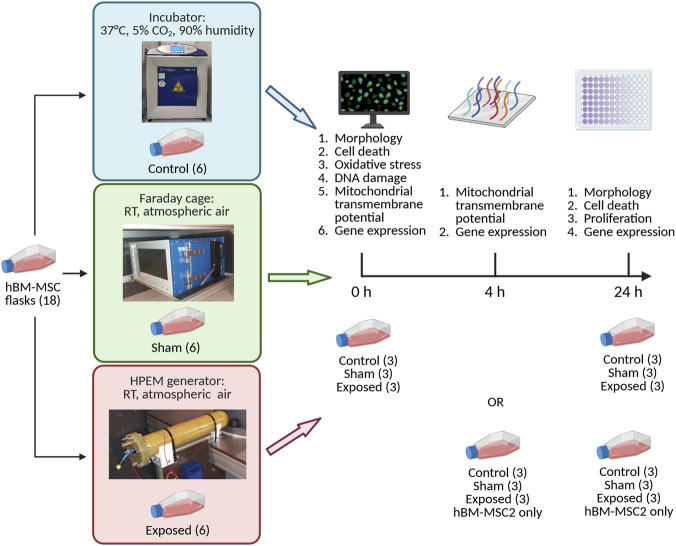
Design of a single experiment. Human bone marrow-derived mesenchymal stem cells (hBM-MSCs) from the same passage were seeded into 18 cell culture flasks. The flasks were then randomly assigned to one of the three groups: control (6 flasks), sham (6 flasks), or exposed (6 flasks). The control cells were maintained under optimal conditions of 37 °C, 5% CO_2_, and 90% humidity. Inside the UCC-L Frankonia anechoic chamber, the sham cells were placed in a Faraday cage, and the exposed cells were placed under a stripline antenna connected to a Marx generator. The sham and exposed cells were kept at room temperature in atmospheric air. After exposure, the control, sham, and exposed cells were harvested immediately (0 h) or after culture in optimal conditions for 24 h. The indicated cellular and molecular processes were then analyzed in three technical replicates. There were two identical experimental replicates for hBM-MSC1. In one of the two experiments hBM-MSC2 cells were harvested after 4 h instead of 0 h. Created in https://BioRender.com.

To analyze the biological effects of a single high-power electromagnetic pulse on human cells, we used an exposure system for generating high-energy electromagnetic pulses which was described in detail by Czwartos and colleagues (Czwartos et al., 2023). Briefly, a 12-stage, 600 kV Marx generator was utilized to deliver a high-magnitude electromagnetic pulse through a 100 Ω stripline antenna. The entire apparatus was mounted on a mobile platform, featuring a central stage for biological samples and a secondary shielded compartment beneath the tabletop for unexposed sham controls. Two cell lines of normal human bone marrow-derived mesenchymal stem cells (hBM-MSCs) from different donors were used for experiments; they are denoted as hBM-MSC1 and hBM-MSC2. They were exposed to a single pulse with a peak field of 1 MV/m, pulse width of 120 ns, and center frequency of 50 MHz. The total exposure time for each sample was limited to the duration of a single 120 ns pulse. For the exposure, the cells were kept attached to the surface of the culture vessels and covered with a minimal volume of cell medium (E, exposed samples). No thermal effects were present during exposure (Czwartos et al., 2023). Sham-exposed cells (S) were processed together with exposed samples, but kept in a Faraday cage during exposure, and used as the main control. In addition, control cells (C) were maintained under optimal conditions throughout the experiment to exclude any potential effects unrelated to the treatment itself (Figure 1).

**FIGURE 2:**
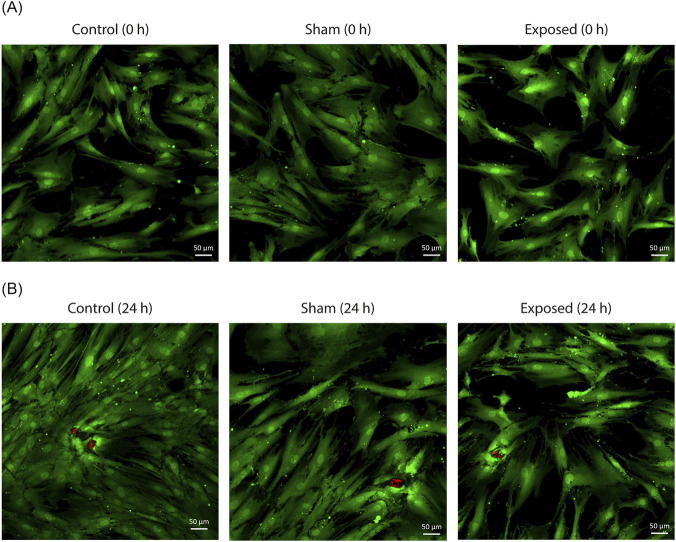
Viability of human bone marrow-derived mesenchymal stem cells (hBM-MSCs) exposed to a single HPEM pulse, sham-exposed, or control. hBM-MSCs were stained for 15 min with the LIVE/DEAD Viability/Cytotoxicity kit **(A)** immediately or **(B)** 24 h after exposure and observed using a confocal microscope (LSM 700 Axio Observer. Z1 Zeiss). Live cells are stained green and dead cells are stained red. Representative images of hBM-MSC2 cell line are shown. Magnification: 100×; bar represents 50 µm.

To assess cellular morphology directly or 24 h after exposure, hBM-MSCs were kept attached to the culture surface, stained with the LIVE/DEAD™ Viability/Cytotoxicity Kit to distinguish between live and dead cells, and visualized with a confocal microscopy. Regardless of the treatment, live cells maintained a typical elongated, spindle-shaped morphology with multiple processes (Figure 2), which is characteristic for a steady-state hBM-MSC culture at early passages (Wagner et al., 2010). More detailed imaging with scanning electron microscopy showed that both exposed hBM-MSC cell lines exhibited normal morphology with multiple processes and numerous microvilli on their surface, similar to sham-exposed and control cells (Supplementary Figure S1).

To evaluate the potential impact of a single electromagnetic pulse on the initial stages of cellular attachment and spreading, hBM-MSC were harvested and imaged under a light microscope 1 hour after seeding. At this early point, the cells predominantly exhibited a circular, spread morphology (Supplementary Figure S2) rather than the polarized, spindle-shaped phenotype seen in Figure 2. This circular appearance represents the initial radial spreading phase, where hBM-MSCs expand uniformly across the surface, as documented in literature (Banik et al., 2016; Long et al., 2019; Larin et al., 2025). Exposure to a single electromagnetic pulse did not affect the cells’ transition from a suspension state to a fully adhered phenotype and did not affect the initial stages of cellular attachment to the surface.

### 3.2 hBM-MSCs viability

The viability of hBM-MSCs was assessed immediately and 24 h after exposure. Staining with the LIVE/DEAD™ Viability/Cytotoxicity Kit revealed that the majority of cells were healthy (green staining) regardless of treatment and time after the experiment (Figure 2). Only a few cells in each condition stained red, indicating damaged cell membranes, but the number of dead cells was similar in control, sham-treated, and exposed samples (Figure 2A).

**FIGURE 3:**
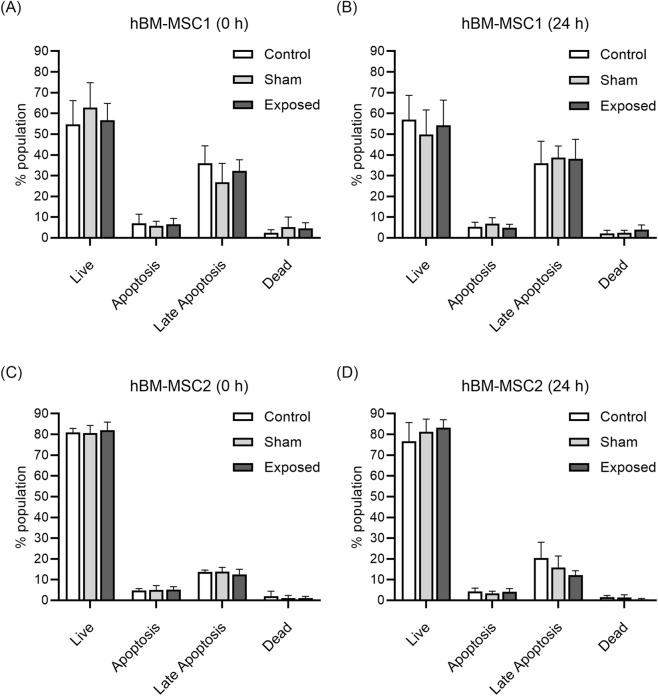
Analysis of cell death in human bone marrow-derived mesenchymal stem cells (hBM-MSCs) exposed to a single HPEM pulse, sham-exposed or control. Cells were stained with Alexa Fluor 488-conjugated Annexin V (AnV) and propidium iodide (PI) immediately or 24 h after treatment. Stained cells were analyzed on a BD FACSAria III flow cytometer with a minimum of 10,000 cells per sample. The graphs show pooled results from two independent experiments with three technical replicates per experiment for **(A)** hBM-MSC1, 0 h post-exposure **(B)** hBM-MSC1, 24 h post-exposure **(C)** hBM-MSC2, 0 h post-exposure **(D)** hBM-MSC2, 24 h post-exposure. Populations are defined as follows: Live (AnV-PI-), Apoptosis (AnV + PI-), late apoptosis (Anv + PI+), dead (AnV-PI+). Each bar represents the mean (±SD).

Detection of cell death by flow cytometry after Annexin V (AnV) and propidium iodide (PI) staining did not reveal any measurable changes in the proportion of cells undergoing apoptosis (AnV positive, PI negative) in exposed samples compared to sham or control samples, both immediately after treatment and after the cells had recovered in normal culture conditions for 24 h (Figure 3). Only a small proportion of cells in all samples were necrotic (AnV-negative, PI-positive), a maximum value of 5.2% ± 4.8% recorded for hBM-MSC1 (sham, 0 h) and 2.0% ± 2.4% for hBM-MSC2 cells (control, 0 h). These results were consistent with LIVE/DEAD Viability/Cytotoxicity Kit staining, which showed a minimal number of cell with a permeabilized cell membrane. Cells from the hBM-MSC1 line (Figures 3A,B) had lower overall viability (AnV-negative, PI-negative) and a higher percentage of cells in late apoptosis (AnV-positive, PI-positive) compared to cells from hBM-MSC2 line (Figures 3C,D), regardless of treatment.

### 3.3 Cell proliferation and metabolic activity changes in hBM-MSCs

**FIGURE 4:**
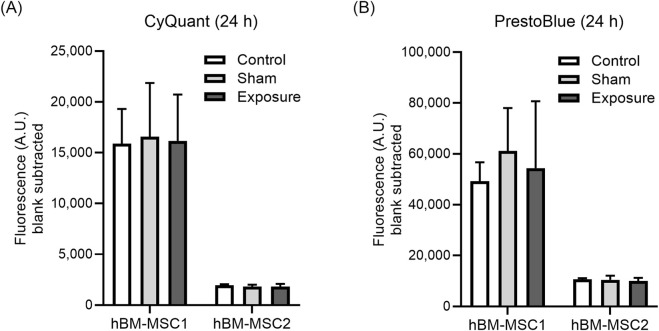
Proliferation of human bone marrow-derived mesenchymal stem cells (hBM-MSCs) exposed to a single HPEM pulse, sham-exposed or control. 24 h after treatment, both hBM-MSC cell lines were stained with **(A)** CyQuant for 1 h or **(B)** Presto Blue for 2 h. Fluorescence intensities were recorded using a CLARIOstar microplate reader (BMG LABTECH). Each graph shows the mean fluorescence readings (±SD), after blank subtraction, from two independent experiments; the number of technical replicates was three for each experiment.

Cell proliferation within the first 24 h after treatment was estimated using a direct method of cellular DNA staining with CyQuant and an indirect method of assessing cell metabolic activity with the resazurin-based PrestoBlue Cell Viability Reagent (Figure 4). Statistical analysis revealed that under the tested conditions the type of treatment had no effect on cells’ proliferation rate, as observed by both CyQuant (Figure 4A) and PrestoBlue staining (Figure 4B). The mean relative fluorescence intensity values (±SD) in CyQuant assay for control, sham and exposed hBM-MSC1 cells were 15886 ± 3429, 16567 ± 5300 and 16167 ± 4562, respectively. Human BM-MSC2 showed consistently lower values in control (1948 ± 117), sham (1835 ± 180) and exposed cells (1824 ± 276) (Figure 4A). In Presto Blue assay, mean relative fluorescence intensity values (±SD) for hBM-MSC1 cells were as follows: control 49202 ± 7499, sham 61134 ± 16855, exposed 54430 ± 26233. For control, sham and exposed hBM-MSC2 cells they were 10717 ± 382, 10475 ± 1590 and 10015 ± 1248, respectively (Figure 4B). As indicated, the 2 cell lines exhibited substantially different baseline signal intensities: 90.1% (p = 0.045) and 83.5% (p = 0.065) of total variation was attributed to the differences between cell lines in CyQuant and Presto Blue assays, respectively.

### 3.4 hBM-MSCs mitochondrial membrane depolarization

Mitochondrial transmembrane potential (ΔΨm) was measured solely in hBM-MSC2 cells as the ratio of JC-1 PE (590 nm)/JC-1 FITC (530 nm) fluorescence intensity and then expressed as a percentage of the sham sample result (Supplementary Figure S3). Mitochondrial membrane depolarization was observed only in positive controls with the ionophore valinomycin, but not in exposed samples. The ΔΨm values measured immediately and 4 h after exposure were reduced in valinomycin controls compared to sham (1.54% ± 0.70% and 31.05% ± 15.16%, respectively). Mitochondrial membrane depolarization was not observed in exposed samples when the test was performed immediately after exposure (99.62% ± 6.37%). A slight decrease in mitochondrial membrane potential was observed in samples 4 h after exposure (85.44% ± 16.73% of sham).

### 3.5 The level of oxidative stress in hBM-MSCs

**FIGURE 5:**
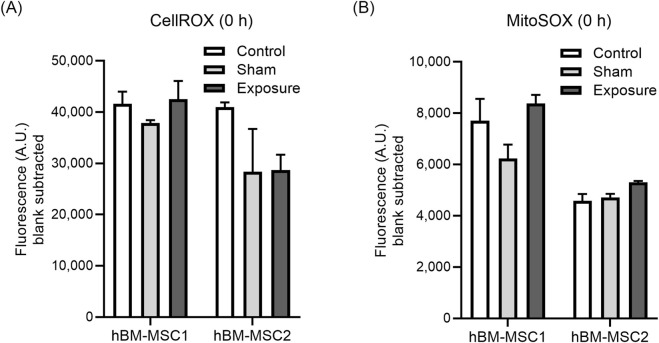
Oxidative stress in human bone marrow-derived mesenchymal stem cells (hBM-MSCs) exposed to a single HPEM pulse, sham-exposed or control. After 30 min of treatment, hBM-MSCs were stained with **(A)** CellROX Orange for 30 min or **(B)** MitoSOX Red for 10 min. Fluorescence intensities were recorded using a CLARIOstar microplate reader (BMG LABTECH). Each graph shows mean fluorescence readings (±SD), after blank subtraction, from two independent experiments with three technical replicates per experiment.

Oxidative stress in hBM-MSCs after treatment was measured with two fluorogenic probes: CellROX Orange, which is activated by cellular reactive oxygen species, and MitoSOX Red, which specifically targets mitochondria and is oxidized by mitochondrial superoxide. Immediately after exposure hBM-MSC1 cells showed mean CellROX Orange fluorescence intensity (42510 ± 3567) comparable to that of control cells (41582 ± 2410). Sham-exposed cells showed a slightly lower signal of 37858 ± 570 (Figure 5A). For hBM-MSC2, both exposed (28705 ± 2992) and sham-exposed cells (28376 ± 8354) showed a similar mean CellROX signal, which was considerably lower than that of control cells (40946 ± 939) (Figure 5A). Statistical analysis indicated differences in CellROX signal intensities between hBM-MSC1 and hBM-MSC2 cells (p = 0.017), whereas the treatment did not impact the cytoplasmic reactive oxygen species levels.

Mitochondrial superoxide production, measured via MitoSOX fluorescence, increased following electromagnetic pulse exposure in both cell lines, with mean signal (±SD) recorded at 8379 ± 332 for hBM-MSC1 (34.3% increase versus sham) and 5298 ± 59 for hBM-MSC2 (12.5% increase versus sham) (Figure 5B). Two-way RM ANOVA revealed the main effect of treatment (p = 0.019). Although baseline fluorescence varied between cell lines (p = 0.024), a consistent trend of increased oxidative stress in the exposure group compared to sham-control (hBM-MSC1: 6236 ± 544, hBM-MSC2: 4708 ± 144), and control (hBM-MSC1: 7709 ± 849, hBM-MSC2: 4581 ± 271) was observed across all independent experiments. Due to the small sample size, Tukey’s post-hoc analysis revealed no statistically significant differences in pair-wise comparisons between the treatment groups.

### 3.6 DNA damage in hBM-MSCs

**FIGURE 6:**
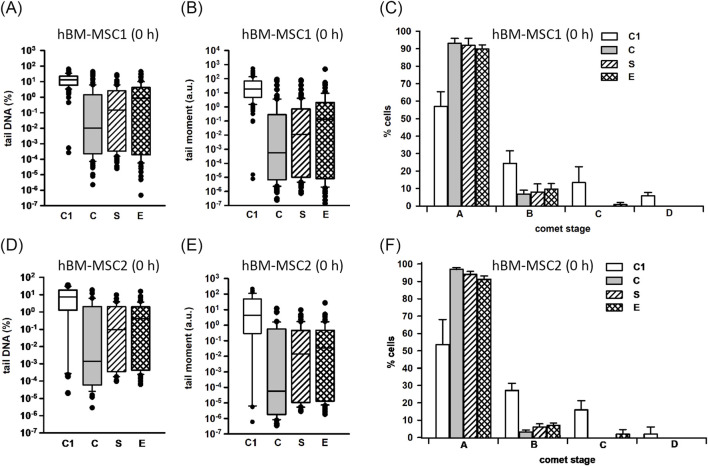
DNA damage in human bone marrow-derived mesenchymal stem cells (hBM-MSC) exposed to a single HPEM pulse, sham-exposed or control. The extent of DNA damage in hBM-MSCs from both cell lines was assessed by the neutral comet assay 30 min after exposure. Abbreviations: C: negative control (unexposed cells); S: sham-exposed cells; E: HPEM-exposed cells; C1: positive control (cells treated with 50 µM etoposide for 24 h) **(A,B,D,E)** DNA damage was expressed as percentage of tail DNA for **(A)** hBM-MSC1 and **(D)** hBM-MSC2, or tail moment for **(B)** hBM-MSC1 and **(E)** hBM-MSC2. Each box shows the median value, represented by a horizontal line within a box, of two independent experiments. The 25^th^ and 75^th^ percentiles are indicated by the bottom and top of the boxes, respectively. Whiskers: 5^th^ and 95^th^ percentiles **(C,F)** Percentages of cells in comet stage categories for **(C)** hBM-MSC1 and **(F)** hBM-MSC2. Stage A cells with intact nuclear DNA; stages B, C, D cells with low, moderate, and high levels of nuclear DNA damage, respectively. Each bar represents the mean (±SD) of two independent experiments.

Double-strand DNA breaks were assessed using the neutral comet assay 30 min after exposure to HPEM pulses (Figure 6). In hBM-MSC1, the median percentage of damaged DNA present in the comet tail was greater in the exposed samples than in the sham samples, recording values of 0.79% and 0.5%, respectively. Nonetheless, this difference did not reach statistical significance (p ≥ 0.05). Meanwhile, the median percentage of tail DNA for the negative control was less than 0.01%, while the positive control (cells treated with etoposide) was at 19.11% (Figure 6A). Similarly, no statistically significant differences were observed in the median tail moment values between the exposed and sham samples (p ≥ 0.05), with values of 0.11 and below 0.01 arbitrary units (a.u.), respectively. The negative and positive controls displayed median tail moments of below 0.01 and 13.12 a. u., respectively (Figure 6B). Nuclear DNA damage was further evaluated using a classification system that includes five comet stages. Stage A indicates cells with intact nuclear DNA, while stages B, C, and D reflect low, moderate, and high levels of DNA damage, respectively. Stage E denotes cells with complete degradation of nuclear DNA, which was not found in this study. A limited number of comets in stage C were noted in the exposed samples, whereas only stages A and B were present in the sham samples. However, the difference in the distribution of comet stages was not statistically significant (Figure 6C).

In line with the results obtained for hBM-MSC1, there was no difference in the median percentage of damaged DNA in the comet tail between the exposed and sham samples for hBM-MSC2 (p ≥ 0.05). The observed percentages were 0.41% for the exposed samples and 0.09% for the sham samples. Concurrently, the median percentage of tail DNA was less than 0.01% for the negative control and 9.20% for the positive control (cells treated with etoposide) (Figure 6D). The median tail moment values did not differ significantly (p ≥ 0.05) between the exposed and sham samples, registering 0.03 and 0.01 arbitrary units (a.u.), respectively. Furthermore, the median tail moment for the negative and positive controls was found to be less than 0.01 and 6.13 a. u., respectively (Figure 6E). Additionally, HPEM exposure did not significantly increase the proportion of cells classified in comet stages B and C when compared to the sham-exposed cells (Figure 6F).

### 3.7 Transient upregulation of cell cycle and oxidative phosphorylation-related gene sets in hBM-MSCs

Gene expression in both hBM-MSC lines was analyzed immediately after exposure, 4 h post-exposure (only for hBM-MSC2), and 24 h post-exposure to a single HPEM pulse. Principal component analysis (Supplementary Figure S4) showed clear clustering of samples based on the cell line. As a result, subsequent analyses were performed separately for each cell line to focus on potential exposure effects rather than individual differences between them. The PCA did not reveal any noticeable differences between exposed, control, and sham-treated cells. Consistently, no genes showed markedly altered expression when comparing the mean gene expression between exposed and sham-control samples at each time point, using a moderated t-test with Benjamini-Hochberg FDR multiple testing correction (Supplementary Figure S5). However, considerable differences in gene expression were observed between both exposed and sham-exposed cells compared to control cells (data not shown), emphasizing the importance of including sham controls in such experiments.

**FIGURE 7:**
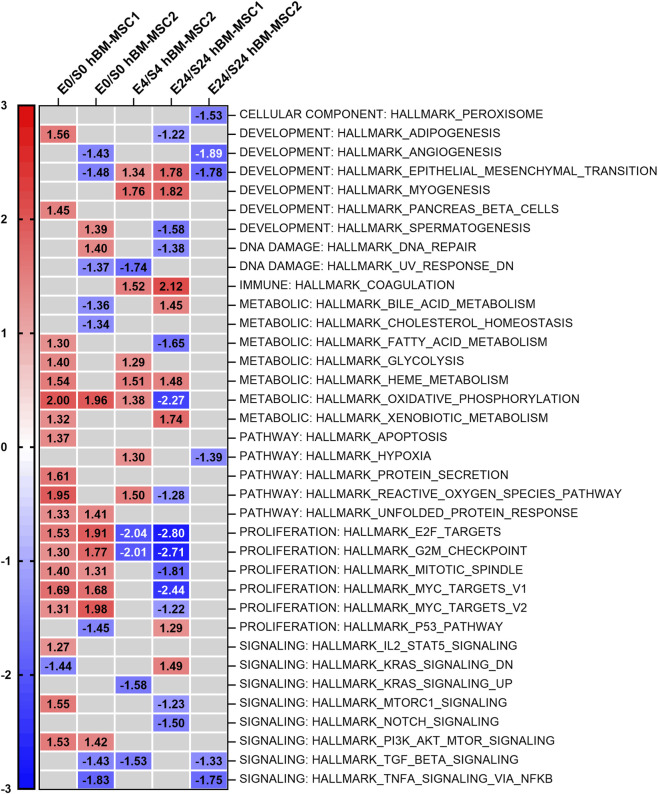
Positively and negatively enriched gene sets in human bone marrow-derived mesenchymal stem cells (hBM-MSCs) exposed to a HPEM pulse compared to sham-exposed hBM-MSCs. Gene set enrichment analysis (GSEA) was performed for exposed and sham-treated hBM-MSC sample pairs at different time points, separately for each cell line. GSEA with a false discovery rate (FDR) and a cutoff of 0.25 was performed using GSEA software v 4.2.3, and hallmark gene sets from the Molecular Signatures Database. The obtained Normalized Enrichment Scores (NES) were presented on a heatmap generated using GraphPad Prism v 10. Hallmark gene sets were grouped by a process category [30]. Red color indicates NES > 0 (positive enrichment, FDR<0.25), blue color indicates NES<0 (negative enrichment, FDR<0.25), grey color indicates results with FDR > 0.25. E0: exposed at 0 h; S0: sham-exposed at 0 h; E4: exposed at 4 h; S4: sham-exposed at 4 h; E24: exposed at 24 h; S24: sham-exposed at 24 h.

The single gene approach may be at a disadvantage when changes in gene expression are subtle but coordinated and affect functionally related gene sets (Maleki et al., 2020). Therefore, we used GSEA software to assess whether the expression of predefined hallmark gene sets from the Human Molecular Signatures Database (MSigDB) (Liberzon et al., 2015) differed between exposed and sham-exposed cells. GSEA identified gene sets, listed in Supplementary Tables S1-S9, that showed positive or negative enrichment in exposed samples with False Discovery Rate below 0.25. Figure 7 shows a heatmap summarizing the Normalized Enrichment Scores (NES) for these 36 hallmark gene sets, grouped by category. The gene sets associated with proliferation, particularly those involved in cell cycle progression (E2F targets, G2/M checkpoint, mitotic spindle), showed the most consistent changes. These gene sets were positively enriched in exposed samples from both cell lines immediately after exposure. However, a negative enrichment effect was observed in hBM-MSC1 at 24 h and in hBM-MSC2 at 4 h (E2F targets and G2M checkpoint gene sets) or only in hBM-MSC1 at 24 h (mitotic spindle, MYC targets V1, MYC targets V2). Among all proliferation-related gene sets, only the P53 pathway did not show a clear pattern of changes. The PI3K-AKT-mTOR pathway and the oxidative phosphorylation gene set were both positively enriched immediately after exposure in both cell lines. The oxidative phosphorylation gene set showed the most substantial changes in terms of Normalized Enrichment Score at the 0-h time point (NES = 2.00 for hBM-MSC1 and NES = 1.96 for hBM-MSC2). It was also positively enriched 4 h after exposure in hBM-MSC2 (NES = 1.38). However, there was a notable negative enrichment of this gene set 24 h after exposure in hBM-MSC1 (NES = 2.27). Reactive oxygen species pathway was also positively enriched in hBM-MSC1 immediately after exposure (NES = 1.95) and in hBM-MSC2 4 h after exposure (NES = 1.50). GSEA results for the other gene sets were inconsistent between cell lines.

To determine whether the enrichment of gene sets in both hBM-MSC lines was due to differences in gene expression within the same gene pool, we performed a leading-edge gene analysis. Supplementary Table S10 shows the list of all identified leading-edge genes with their corresponding Signal2Noise scores. The Signal2Noise score represents the difference between the gene expression means in exposed and sham-exposed cells, scaled by the pooled standard deviation. Genes were compared between the 2 cell lines at each time point, separately for up- and downregulated genes, with overlapping genes found in two conditions. Genes overexpressed in exposed cells compared to sham-exposed cells immediately after exposure showed the highest overlap between hBM-MSC1 and hBM-MSC2 cell lines. This group consisted of 266 genes, which are listed in Supplementary Table S11. The eight genes downregulated at 24 h in both cell lines included SMAD3 (SMAD family member 3), JAG1 (jagged canonical Notch ligand 1), NOTCH2 (notch receptor 2), ACAA1 (acetyl-CoA acyltransferase 1), RETSAT (retinol saturase), CRAT (carnitine O-acetyltransferase), CAVIN1 (caveolae associated protein 1), and MARCKS (myristoylated alanine-rich protein kinase C substrate).

**FIGURE 8:**
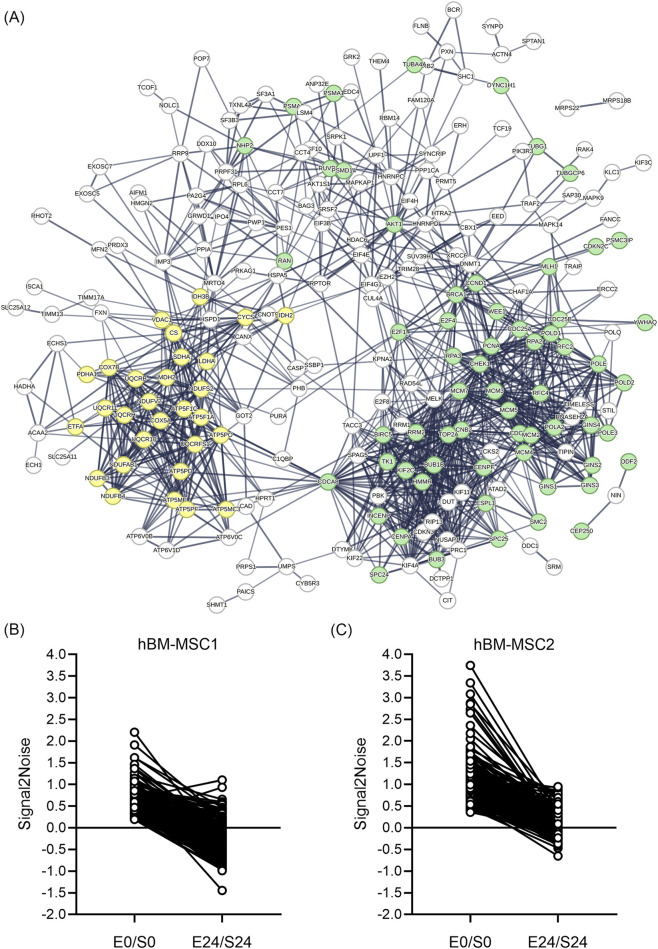
Analysis of 266 leading-edge genes, common for both hBM-MSC cell lines, from gene sets positively enriched in HPEM-exposed versus sham-treated hBM-MSCs at time 0 h. **(A)** Leading-edge gene network generated with STRING. Type of network: full STRING network; nodes: genes; edges: confidence of interaction; minimum required interaction score: 0.7 (high confidence); disconnected nodes not visible. The two tightly connected gene clusters contain genes involved in the cell cycle (Reactome HSA-1640170, colored green) and genes from the citric acid (TCA) cycle and respiratory electron transport chain (Reactome HSA-1428517, colored yellow). **(B–C)** Plots of Signal2Noise (the difference of gene expression means between HPEM-exposed and sham-treated hBM-MSCs scaled by the pooled standard deviation) calculated in GSEA for leading-edge genes common for both hBM-MSC cell lines at time 0 h **(B)** results for hBM-MSC1: Signal2Noise scores at 0 h and 24 h **(C)** results for hBM-MSC2: Signal2Noise scores at 0 h and 24 h.

Subsequent gene network analysis was performed on common genes overexpressed in exposed cells immediately after exposure using STRING, which allowed the identification of two distinct clusters (Figure 8A). One cluster consisted mainly of genes involved in the cell cycle (Reactome HSA-1640170, false discovery rate 2.66E-32; colored green in Figure 8A), while the other cluster contained genes related to the citric acid (TCA) cycle and the respiratory electron transport chain (Reactome HSA-1428517, false discovery rate 1.03E-18; colored yellow in Figure 8A). The latter contained primarily the components of mitochondrial NADH:ubiquinone oxidoreductase (respiratory complex I), ubiquinol-cytochrome c oxidoreductase (respiratory complex III), and mitochondrial membrane ATP synthase (F (1)F (0) ATP synthase). However, the initial effect of overexpression of these gene groups in exposed cells compared to sham cells was diminished after 24 h. This was evidenced by the decrease in Signal2Noise values (Figures 8B,C). In the case of hBM-MSC1, the early overexpression of the top 266 genes was lower than in hBM-MSC2, and the decrease in their expression after 24 h corresponded to a considerable negative enrichment in most of the proliferation-related and oxidative phosphorylation gene sets (Figure 7).

## 4 Discussion

In this preliminary study, we exposed two human bone marrow-derived mesenchymal stem cell lines to a single wideband high-power electromagnetic pulse with a peak field of 1 MV/m, pulse width of 120 ns and a center frequency of 50 MHz to simulate a realistic human exposure to extremely high field strengths. The specific aim was to characterize the acute and early biological response of hBM-MSCs to this singular, high-energy event. To capture the full kinetic profile of the cellular response, moving from immediate physical trauma to delayed biochemical signaling, we selected three distinct observation windows (Figure 1). The immediate post-exposure window (0 h) was used to assess acute physical effects on cellular morphology and plasma membrane integrity, cell viability, reactive oxygen species production, DNA damage, as well as initial shifts in mitochondrial transmembrane potential and gene expression (immediate-early response genes (Bahrami and Drabløs, 2016)). The 4-h point served as a transition phase to monitor secondary transcriptional stress and mitochondrial shifts. Finally, the 24-h window was selected to assess stabilized outcomes, such as sustained changes in morphology and membrane integrity, definitive commitment to apoptosis/necrosis, broader metabolic and proliferative shifts, and sustained changes in gene expression. By tracking these parameters over 24 h, we aimed to distinguish between transient stress and sustained functional impairment following a single HPEM. It also allowed us to compare our results with a previous work on the effects of a single HPEM pulse on hBM-MSCs by using the same time points (Czwartos et al., 2023).

The primary finding of this study is that a single high-power electromagnetic pulse does not compromise the long-term biological integrity or viability of human bone marrow-derived mesenchymal stem cells (hBM-MSCs). Our results demonstrate that exposed cells maintain normal morphology (Figure 2; Supplementary Figures S1-S2) and do not undergo excessive apoptosis or necrosis (Figures 2, 3). Furthermore, critical indicators of cellular health, including DNA integrity (Figure 6) and mitochondrial transmembrane potential (Supplementary Figure S3), remained unaffected. The most notable biological responses were transient and focused on mitochondrial metabolism. We observed a consistent, but relatively small increase in mitochondrial superoxide production, suggesting a temporary surge in oxidative stress (Figure 5B). While individual gene expression remained stable, gene set enrichment analysis (GSEA) revealed a coordinated but temporary upregulation of pathways related to the cell cycle and oxidative phosphorylation (Figure 7). Notably, we identified a core set of 266 genes common to both donors that were upregulated immediately following exposure before returning to baseline, suggesting a robust but reversible molecular response to HPEM (Figure 8). To our knowledge, this is the first comprehensive characterization of broadband HPEM pulse effects on the cellular and molecular landscape of human bone-marrow derived MSCs.

The present study has two notable strengths. First, it used bone marrow-derived mesenchymal stem cells from two independent donors. To reduce the sources of heterogeneity (Česnik and Švajger, 2024), both primary hBM-MSC lines were obtained from the same commercial source, which uses identical, highly standardized protocols for characterization and quality control, in line with recommendations from the International Society for Stem Cell Research (Ludwig et al., 2023). Moreover, both cell lines were isolated from bone marrow of young donors (ages 22 and 24), expanded under the same conditions in our laboratory and treated at the fourth or fifth passage. Consistent with previous findings (Siegel et al., 2013), hBM-MSCs from the two donors showed some intrinsic differences under experimental conditions. For instance, hBM-MSC1 cells presented decreased viability compared to hBM-MSC2 cells, regardless of treatment (Figure 3). A clear variability in the overall gene expression pattern was also observed between these 2 cell lines in PCA analysis (Supplementary Figure S4). Despite the differences, the primary biological trends and specific gene set responses remained consistent across both cell lines. Moreover, this study identified a reproducible 266-gene expression signature associated with HPEM exposure, despite the described differences in hBM-MSC transcriptomes and proteomes from different donors (Mindaye et al., 2013; Zhang et al., 2021). While we acknowledge that the statistical power for population-level inferences is inherently limited with a sample size of 2 cell lines, the consistency between them suggests that the observed effects are a robust feature of hBM-MSC biology rather than a donor-specific artifact. A further strength of this study was the use of a single batch of cells for all assays within each experiment. This approach minimized technical variation and controlled for the biological drift often associated with different passages of primary stem cells (Ludwig et al., 2023).

The primary limitations of the study are the exposure of hBM-MSCs to a single HPEM pulse, analysis of short-term effects only (up to 24 h) and a limited number of biological replicates. The first limitation was inherently imposed by the design of the experiment, which simulated a real-life scenario of an electromagnetic exposure with a non-repetitive source capable of generating a high-power pulse. Nevertheless, our observation of stable cell proliferation is consistent with the work of Gibot and colleagues (Gibot et al., 2019), whose experimental design included numerous pulses. They found no differences in the growth of spheroids formed from normal human fibroblasts or colon carcinoma cells HCT-116 when exposed to 2500 pulses with a peak field of 190 kV/m (pulse width 15 ns, center frequency 195 MHz) or 2500 and 5000 pulses with a peak field of 72.5 or 172.5 kV/m (pulse width 15 ns, center frequency 150 MHz). On the other hand, a study using moderate-intensity UWB exposure of pre-neoplastic CL-S1 mammary epithelial cells (electric field strength of 18 kV/m, pulse width of 10 ns, pulse rise of 0.1 ns) showed that prolonged treatment for more than 4 h with electromagnetic pulses at 1 kHz and 10 kHz pulse repetition rate increased cell growth. This effect was not observed with shorter exposure times (Sylvester et al., 2005). One could speculate that a higher number of pulses in our experimental setting may have a more pronounced effect on hBM-MSC, although this remains to be determined.

The limited observation time was necessitated by the biological model. Bone marrow-derived mesenchymal stem cells have a limited lifespan and undergo senescence after six to eight passages in long-term culture (Wang et al., 2021). Results obtained from longer observations may be affected by these changes. After propagating the cells to achieve sufficient numbers for the experiments, hBM-MSCs were already at the fourth or fifth passage, leaving no margin for long-term observations. However, the lack of immediate genotoxic effect, no observed changes in cell morphology and viability suggest that it is less likely that long-term effects are important. The effect of HPEM pulses on stem cell differentiation was not the subject of this study.

We acknowledge that our study is characterized by a limited number of biological replicates (n = 2), which naturally restricts the broad generalizability of the findings. While a Two-Way Repeated Measures ANOVA with a matching design and Geisser-Greenhouse corrections was utilized to robustly control for donor-to-donor variance and provide conservative statistical inference, the reported p-values should be interpreted as indicators of strong, reproducible trends within the tested primary cell lines rather than absolute universal effects.

The magnitude of the cellular and molecular effects observed in this study after the exposure of hBM-MSCs to the HPEM pulse was relatively modest. They did not appear to permanently affect hBM-MSCs cellular processes.

Increased superoxide production in mitochondria immediately after the exposure (Figure 5B) should be treated as a preliminary observation and interpreted with caution due to the small sample size. Nevertheless, a coordinated overexpression of genes coding for proteins involved in reactive oxygen species inactivation in mitochondria, such as superoxide dismutase 2 (SOD2) or thioredoxin reductase one and 2 (TXNRD1, TXNRD2), was observed in hBM-MSC1 cell line immediately after the exposure to HPEM pulse (Supplementary Table S10). Induction of oxidative stress after exposure to electromagnetic pulses was observed previously in a limited number of animal studies. Mice exposed to UWB pulses with peak field strength of 98 kV/m for 60 min showed increased activities of antioxidant enzymes and increased levels of the lipid peroxidation end product MDA (malondialdehyde) in liver and kidney cells. Exposure for 10 min at a peak field strength of 344 kV/m affected only liver cells (Guo et al., 2020). Similarly, rats exposed to 200 electromagnetic pulses with a peak intensity of 400 kV/m (rise time 10 ns, pulse width 350 ns, 0.5 pps, 1 Hz) showed increased MDA levels and increased superoxide dismutase (SOD) activity (Deng et al., 2014). That being said, a previous study in our laboratory utilized electron paramagnetic resonance (EPR) spectroscopy, a technique with significantly higher sensitivity for radical detection than the fluorogenic probes used here. Despite this increased resolution, EPR measurements performed immediately after HPEM exposure revealed no discernible differences in free radical levels between exposed hBM-MSCs and sham controls (Czwartos et al., 2023).

In this study, we identified changes in gene expression common for both hBM-MSC cell lines. However, they were only detected at the level of gene sets (Figure 7), not individual genes, confirming the modest effect of HPEM pulse on hBM-MSCs. Only transient increase in the expression of genes involved in cell cycle and oxidative phosphorylation was detected. Consistent with this, no changes in cell proliferation (Figure 4) or mitochondrial transmembrane potential (Supplementary Figure S3) were observed. These results suggest that although a single pulse has a transient effect on gene expression, the outcome is not biologically significant. The cells showed the ability to return to their homeostatic balance. Only one previous study investigated gene expression changes in human cells exposed to UWB electromagnetic field pulses with an average peak of 100 kV/m (pulse width 0.79 ± 0.01 ns, pulse repetition rate 50 pps) with no associated thermal effects. Despite increased NFKB binding to DNA, no upregulation of NFKB-dependent target genes was detected at 8 h and 24 h after treatment (Natarajan et al., 2006), consistent with our observations.

The biological effects of high-power ultrawideband (UWB) pulses reported in the literature vary widely due to inconsistent pulse parameters and a lack of standardized reporting on pulse shape and antenna characteristics (Schunck et al., 2014). Furthermore, many studies rely on a narrow range of biological assays. Our work addresses these gaps by providing a multi-scale characterization of the hBM-MSC response to a single 1 MV/m broadband pulse. In conclusion, while this high-intensity pulse triggers an immediate molecular response, hBM-MSCs remain biologically resilient. The primary effects are transient, characterized by a temporary surge in mitochondrial superoxide and the coordinated upregulation of gene sets related to oxidative phosphorylation and the cell cycle. These shifts are reversible and do not translate into long-term cellular impairment; morphology, DNA integrity, and cell viability remained similar to sham-exposed controls.

To build upon these findings and address current limitations, future research should incorporate a larger cohort of primary hBM-MSC lines to improve statistical power and clarify whether the observed oxidative stress results are reproducible. Additionally, testing other cell models is necessary to confirm whether the cellular resilience demonstrated by these stem cells is a consistent trait across different human tissues. Future studies should also focus on uncovering the precise upstream biophysical mechanisms driving this transient interaction, exploring pathways such as plasma membrane permeabilization or direct electromagnetic effects on the mitochondrial electron transport chain. Furthermore, while a single pulse appears safe, investigating the cumulative effects of repetitive HPEM exposure over extended observation periods is essential. Such studies will be critical for evaluating long-term functionality, including differentiation potential and the induction of cellular senescence, ultimately providing the data necessary to update human electromagnetic exposure guidelines.

## Data Availability

The datasets presented in this study can be found in online repositories. The names of the repository/repositories and accession number(s) can be found below: https://www.ncbi.nlm.nih.gov/, GEO Series accession number GSE278937 (https://www.ncbi.nlm.nih.gov/geo/query/acc.cgi?acc&equals;GSE278937).
