# Supplementary material for: Transient biological response of human mesenchymal stem cells to a single wideband high-power electromagnetic pulse exposure: a preliminary study
Source: Front Cell Dev Biol. 2026 Jul 2;14:1873951. doi: 10.3389/fcell.2026.1873951 (PMC13373032; doi:10.3389/fcell.2026.1873951)
Supplement: Supplementary file 1 [file DataSheet1.PDF]

Supplementary Material

1 Supplementary Figures

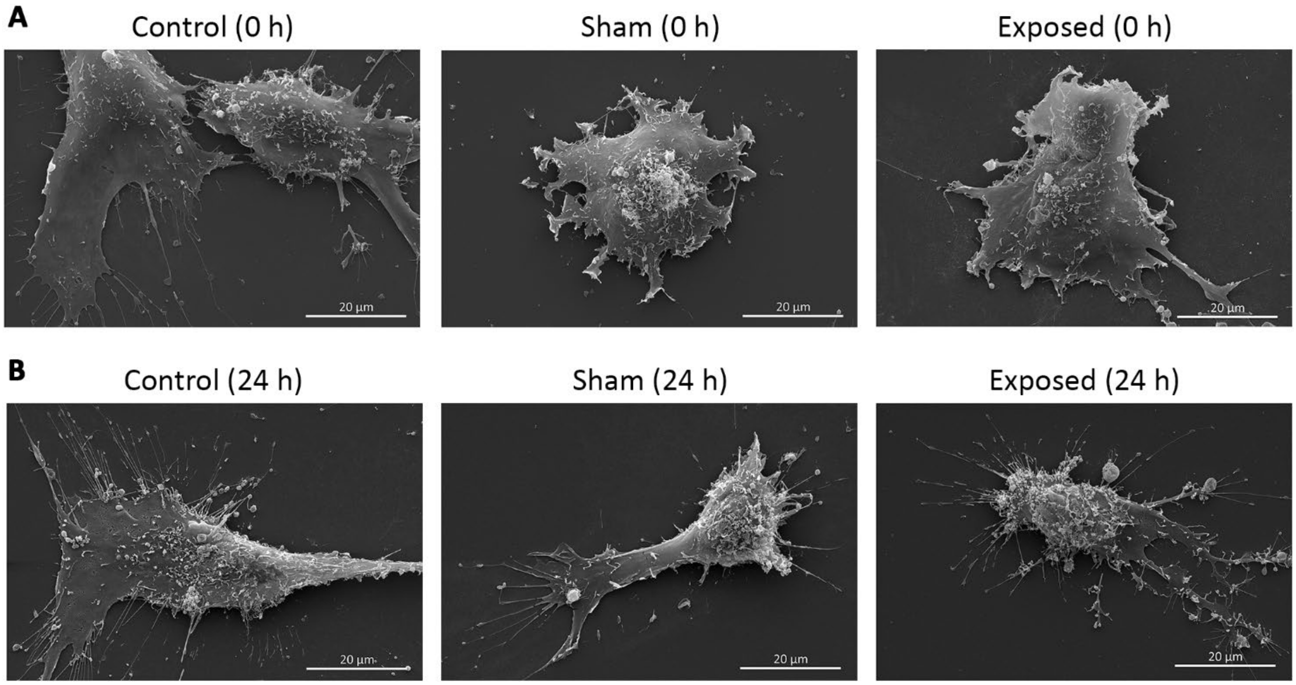

Supplementary Figure 1. SEM images of human bone marrow-derived mesenchymal stem cells (hHBM-MSCs) exposed to a single HPEM pulse, sham-exposed or control. Cells were harvested **(A)** immediately or **(B)** 24 hours after exposure, allowed to adhere for 2 hours before fixing and observed using a scanning electron microscope STEM (Quanta FEG250, FEI) in a high vacuum mode with Everhart-Thornley (ETD) detector. Representative images of hHBM-MSC2 cell line are shown. Magnification: 5000×, the bar represents 20 µm.

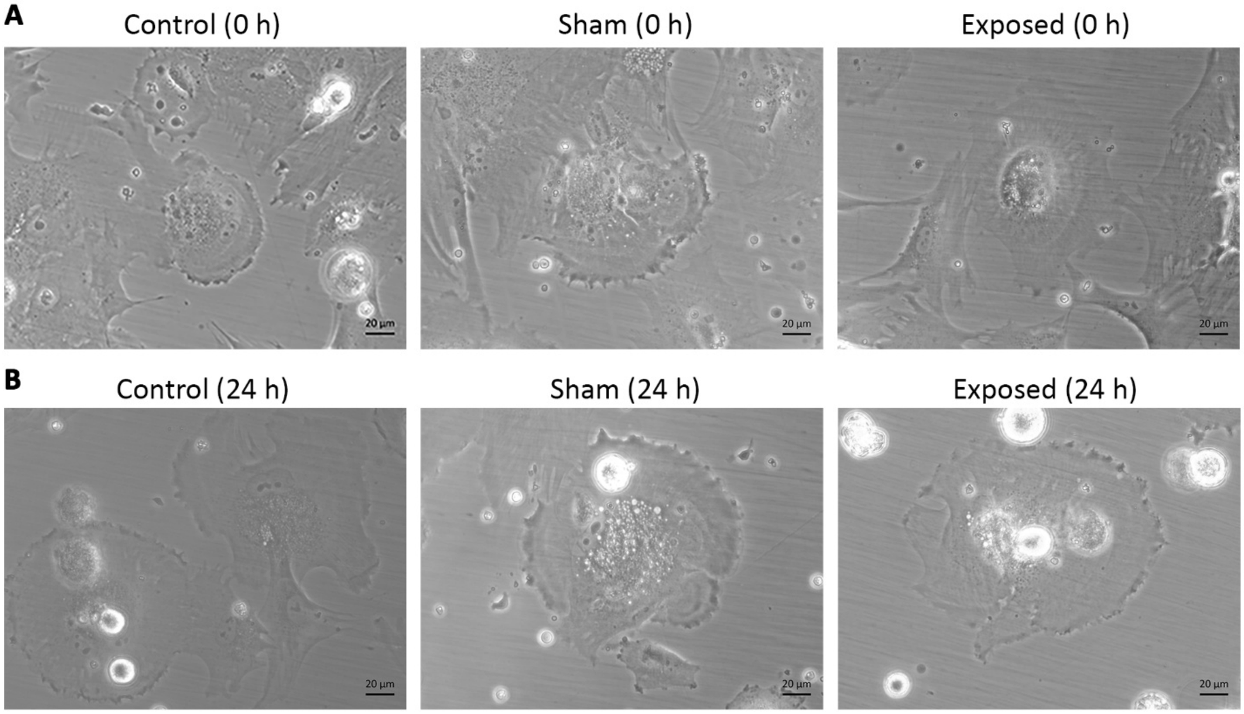

Supplementary Figure 2. Morphology of human bone marrow-derived mesenchymal stem cells (hHBM-MSCs) exposed to a single HPEM pulse, sham-exposed or control at the initial stages of cellular attachment to the surface. Cells were harvested **(A)** immediately or **(B)** 24 hours after treatment and allowed to adhere for 1 hour. Cell morphology was recorded using an inverted optical microscope (Primo Vert, Carl Zeiss) equipped with ZEISS ZEN software (Carl Zeiss). Representative images of hHBM-MSC2 cell line are shown. Magnification: 40×; the bar represents 20 µm.

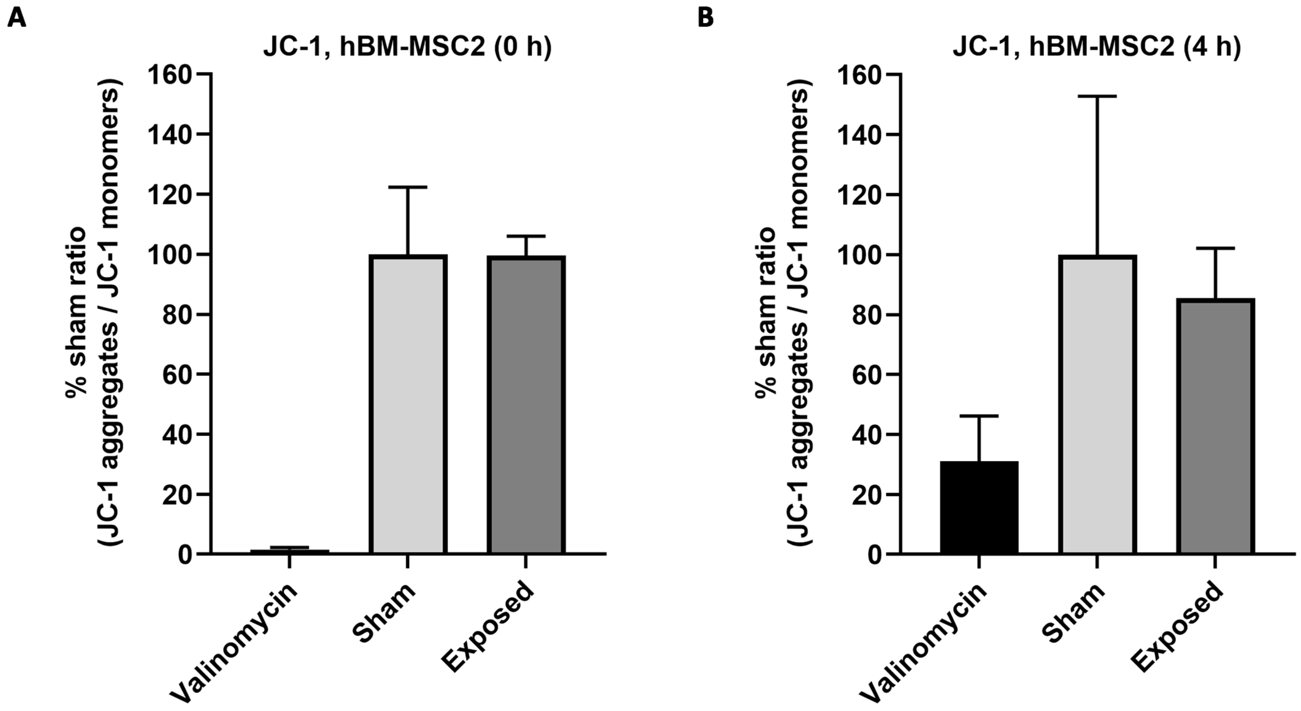

Supplementary Figure 3. Mitochondrial transmembrane potential of human bone marrow-derived mesenchymal stem cells (hHBM-MSCs) exposed to a single high-power electromagnetic pulse and sham-exposed. Cells from the hHBM-MSC2 line were stained with JC-1 dye for 20 min **(A)** immediately or **(B)** 4 hours after the treatment. Valinomycin-treated cells served as a positive control. After staining cells were analyzed on BD FACSAria™ III flow cytometer (at least 10,000 cells per sample). The ratio of yellow to green fluorescence was calculated for all samples. Then, the results were normalized to sham and presented as mean of three technical replicates (±SD).

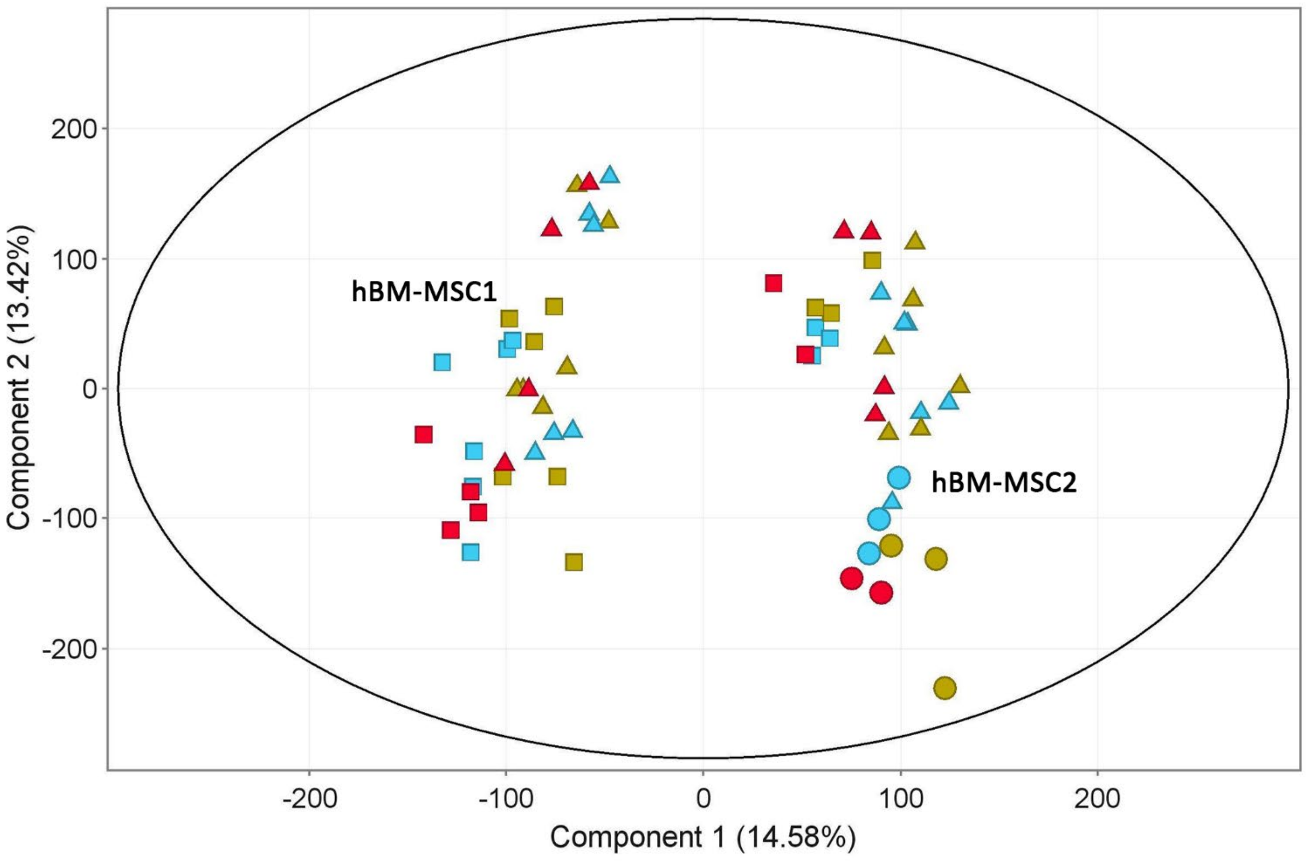

Supplementary Figure 4. Principal component analysis score plot of gene expression in human bone marrow-derived mesenchymal stem cells (hHBM-MSCs) exposed to a single HPEM pulse, sham-exposed or control. Axes: Principal Component 1 (PC1) and Principal Component 2 (PC2) with the respective proportion of variance explained by the component. Points representing individual samples are colored by treatment (red: C - control, blue: S - sham-exposed, yellow: E – exposed) and shaped by time of cells harvesting after treatment (square – 0 h, circle – 4 h, triangle – 24 h). The outer ellipse represents the Hotelling T2 with 99% confidence in the score plot. Separation of samples from two hHBM-MSC cell lines is clearly visible.

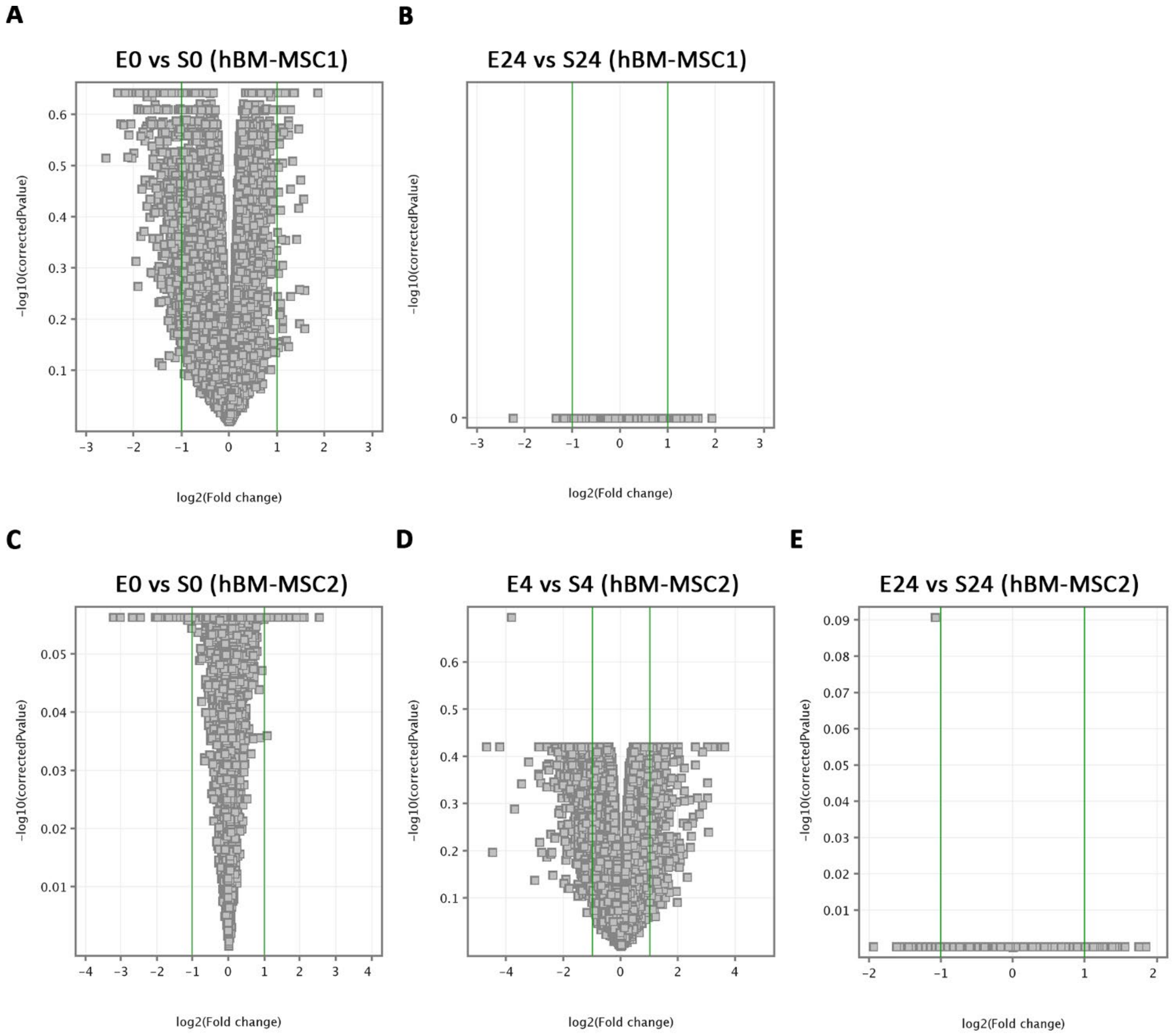

Supplementary Figure 5. Differential gene expression analysis of human bone marrow-derived mesenchymal stem cells (hBM-MSC) exposed to a single HPEM pulse and sham-exposed. Volcano plots compare hHBM-MSCs exposed to a single HPEM pulse versus sham-exposed controls. The x-axis represents the log_2 fold change, and the y-axis represents the $-log_{10}$ of corrected p-values. Statistical significance was determined using a moderated t-test with Benjamini-Hochberg FDR correction. **(A)** hBM-MSC1 exposed (E0) vs sham-treated (S0) at 0 h; **(B)** hBM-MSC1 exposed (E24) vs sham-treated (S24) at 24 h; **(C)** hBM-MSC2 exposed (E0) vs sham-treated (S0) at 0 h; **(D)** hBM-MSC exposed (E4) vs sham-treated (S4) at 4 h; **(E)** hBM-MSC2 exposed (E24) vs sham-treated (S24) at 24 h. Data in **(A)**, **(B)**, and **(E)** represent pooled results from two independent experiments; **(C)** and **(D)** show results from a single experiment.
